# The Level of Knowledge About Antenatal Care Among Women of Childbearing Age in the United Arab Emirates

**DOI:** 10.7759/cureus.99834

**Published:** 2025-12-22

**Authors:** Aya S Aldaher, Kawthar A Almuharraqi, Osama S Alrjoub, Mohamad O Haji Hasan, Rogayah A Abdalhameed, Maethah H Alzarooni, Suni Ebby, Amal Hussein

**Affiliations:** 1 College of Medicine, University of Sharjah, Sharjah, ARE; 2 Basic Medical Sciences, University of Sharjah, Sharjah, ARE; 3 Family and Community Medicine and Behavioral Sciences, Public Health, University of Sharjah, Sharjah, ARE

**Keywords:** antenatal care, childbearing age women, maternal health, pregnancy awareness, pregnancy outcomes, public health, uae, women’s health

## Abstract

Introduction

Antenatal care (ANC) refers to the care provided to pregnant women from conception to childbirth and is strongly associated with improved pregnancy outcomes. However, within the United Arab Emirates (UAE), limited recent data exist to address the level of awareness about ANC among women of childbearing age. This study aims to evaluate the level of practice and depth of knowledge about ANC among this demographic.

Methods

A cross-sectional study was conducted among women in the UAE from different public locations in Sharjah, Dubai, and Ajman. A total of 426 responses were collected using a self-administered questionnaire, available in both Arabic and English, comprising 31 questions. The data included the participants' demographics, practices, attitudes, and general knowledge about ANC. Data was analyzed using Statistical Package for the Social Sciences (SPSS) version 20 (IBM Corp., Armonk, New York, USA).

Results

More than half (55.4%) of the 426 women who participated were students, and 49.8% held a bachelor's degree, while 54.0% were aged between 18 and 23 years old. Overall, 60.3% of the participants had previously heard about ANC, and the majority (94.1%) recognized its importance during pregnancy. Of the 110 women who had been pregnant, 52 (47.3%) reported attending ANC visits. The level of knowledge about ANC varied between good (39%), moderate (45%), and poor knowledge (16%). Higher levels of knowledge were observed in women with college degrees (p = 0.015), women employed in the healthcare sector (p = 0.001), multigravida women (p = 0.04), and women from higher-income families (p = 0.007). Knowledge level showed no association with age or marital status. 50% of the participants were unaware of the recommended dietary habits and weight gain during pregnancy, and only 16.3% correctly identified the proper timing for initiating folic acid intake during pregnancy.

Conclusion

While most participants had moderate and good knowledge about ANC, a significant proportion had never heard of these services. Awareness campaigns are crucial for addressing this gap and ensuring that all women of childbearing age in the UAE are well-informed about the importance of ANC, ultimately contributing to improved maternal and neonatal outcomes.

## Introduction

Pregnancy is an exceptional female experience that leads to various physiological and psychological changes in the female body. However, it can sometimes be accompanied by complications and issues that put the mother's and the fetus's life in danger. The mother's health is closely linked to the well-being of her child. Physical and mental health before, during, and after pregnancy have a significant impact on the infant's overall health.

Antenatal care (ANC) is the care provided for pregnant women from conception to childbirth. This period provides healthcare professionals with opportunities to reach pregnant women and implement interventions that protect both the mother and the baby. Women attend multiple appointments during which they learn from professionals about healthy practices during pregnancy, monitor the fetal growth, screen for infectious diseases or inherited disorders, test for chromosomal abnormalities, assess and manage maternal conditions including gestational diabetes and gestational hypertension, and receive the necessary physical and emotional support. A pregnant woman receiving the recommended ANC services is more likely to have healthier pregnancy outcomes and to have healthier babies [1]. These outcomes include reduced both maternal and fetal mortality and morbidity, lower risk of infections, better control of complications, lower risk of preterm birth and low birth weight, and increased likelihood of spontaneous delivery.

According to the World Health Organization, the leading cause of death and disabilities among women of childbearing age is complications during pregnancy and delivery [2]. In 2015, around 303,000 women lost their lives due to complications related to pregnancy, and about 2.6 million babies were stillborn [3,4]. Many adverse outcomes could have been avoided through access to high-quality antenatal and postnatal care [2].

Every woman, whether experiencing a high- or low-risk pregnancy, requires continuous access to maternal health care. However, in many regions, women face various barriers that prevent them from receiving adequate ANC. These challenges may include distance, financial constraints, time constraints, fear, and cultural barriers. A crucial factor in overcoming these obstacles is ensuring that women have the knowledge and awareness to understand the importance of ANC and how to seek it.

Studies from diverse settings have consistently found that higher knowledge of ANC significantly correlates with improved ANC practices. This underscores that improving women’s knowledge, along with supportive factors such as media exposure, enhances the utilization of maternal health services [5-9].

While health education plays a vital role in enabling women to understand and monitor their well-being during pregnancy, there is, to our knowledge, a lack of recent data on maternal health knowledge in the United Arab Emirates (UAE). Therefore, our study assessed the knowledge and practices towards ANC among women of childbearing age in the UAE.

## Materials and methods

Study design and setting

A quantitative cross-sectional study was conducted over a four-month period, from February to June 2023, to evaluate the knowledge and practices of ANC among women of childbearing age in the UAE. Women between 18 and 35 years of age residing in the UAE who can speak Arabic or English were enrolled in the study. Participants reported whether they could speak Arabic or English, and because the questionnaire was self-administered in these languages, completing it confirmed their eligibility. Participants who speak neither Arabic nor English, non-UAE residents, and those with cognitive disabilities that limited their decision-making and refused to participate were excluded from the study. Participants were selected based on a non-probability convenience sampling technique. Data were collected from public spaces, including malls, parks, and beaches, in Dubai, Sharjah, and Ajman emirates to provide a geographically varied representation in the northern Emirates. A minimum sample size of 385 was calculated based on 5% marginal error and 50% prevalence using the following formula:

$n = \frac{Z^{2} \, p \, (1 - p)}{ME^{2}}$,

where n is the sample size, p is the expected prevalence, ME is the marginal error, and Z is the z-score for 95% confidence level.

Questionnaire development

A structured, self-administered questionnaire was developed after reviewing the relevant literature and previously validated tools used to assess antenatal care (ANC) knowledge and practices. The aim was to include items that covered key ANC domains, such as warning signs in pregnancy, recommended visit schedules, routine investigations, nutrition, and folic acid supplementation.

The questionnaire consisted of 31 items divided into demographics, obstetric history, practices, and knowledge. Multiple-choice, true/false, and Likert-scale formats were used to ensure clarity and ease of completion, as shown in Appendix A.

To ensure content accuracy and relevance, the questionnaire was reviewed by faculty members from the Departments of Public Health and Obstetrics, who provided expert input on the selected questions and recommended modifications. A pilot study was conducted with 20 women to evaluate the clarity, comprehension, and flow of both the Arabic and English versions of the questionnaire. Participants were recruited from the same types of public locations later used in the main study, including Dubai, Sharjah, and Ajman, to ensure representation from the targeted geographical areas. Feedback from the pilot led to minor adjustments in wording and ordering of questions to improve readability and reduce ambiguity. Data from the pilot were not included in the final analysis.

Data collection and analysis

This study received ethical approval from the Research Ethics Committee of the University of Sharjah, reference number: REC-23-02-19-05-S, approval date: 27 February 2023. During data collection, participants were informed that their participation was voluntary and that all responses would only be used for academic purposes. An information sheet was provided indicating agreement to participate in the study. No personal identifying information was collected, ensuring participants remain anonymous. A total of 426 responses were collected from public places in Dubai, Sharjah, and Ajman. The collected data were only accessible by the researchers and discarded upon the study's completion.

The collected data were entered, coded, and analyzed using Statistical Package for the Social Sciences (SPSS) version 20 (IBM Corp., Armonk, New York, USA). Demographic data were summarized using frequency distributions and percentages. The chi-square statistical test was employed to evaluate relationships between different variables and to assess statistical significance. A significant value was defined as a p-value that was less than 0.05 (p < 0.05).

Each question/sub-question in the questionnaire was assigned one point out of 48. The levels of knowledge were categorized as follows: poor knowledge, scores less than 25 (score < 52%); moderate knowledge, scores ranging from 25 to 34 (52% ≤ score ≤ 71%); good knowledge, scores ranging from 35 to 48 (score ≥ 72%).

## Results

Sociodemographic characteristics

A total of 426 participants were included in this study. Table 1 presents the sociodemographic characteristics of the respondents. The majority of participants (54.0%) were aged between 18 and 23 years. Most were Arab residents (71.1%), followed by UAE nationals (21.4%) and non-Arab residents (7.5%). More than half of the respondents were students (55.4%). Regarding education, 49.8% held a bachelor’s degree, and 35.4% had completed high school. The majority were never married (69.7%), and about one-third (31.2%) reported a monthly household income of ≥15,000 AED.

**Table 1:** Sociodemographic characteristics of study participants (n = 426).

Demographic		Frequency (n)	Percentage
Age (in years)	18-23	230	54.00%
	24-29	107	25.10%
	30-35	89	20.90%
Nationality	UAE national	91	21.40%
	Arab resident	303	71.10%
	Non-Arab resident	32	7.50%
Occupation	Student	236	55.40%
	House wife	67	15.70%
	Currently/previously employed in a healthcare sector	41	9.60%
	Currently/previously employed in a non-healthcare sector	50	11.70%
	Unemployed	32	7.50%
Education level	Post-graduate degree	17	4.00%
	Bachelor's degree	212	49.80%
	High school degree	151	35.40%
	Diploma degree	35	8.20%
	Others	11	2.60%
Marital status	Never married	297	69.70%
	Currently married	123	28.90%
	Previously married	6	1.40%
Average monthly income	<4,000 AED	33	7.70%
	4,000-9,999 AED	77	18.10%
	10,000-14,999 AED	65	15.30%
	≥15,000 AED	133	31.20%
	Refused to answer	118	27.70%

Obstetric history and pregnancy knowledge 

Analysis of participants’ obstetrical history showed that out of the 426 participants, 316 women (74.2%) had never been pregnant. Table 2 shows the distribution of obstetrical data for the 110 participants (25.8%) who reported previously being pregnant.

**Table 2:** Obstetrical history among previously pregnant participants (n = 110).

Category	Response	Count (n)	Percentage (%)
Number of pregnancies	One pregnancy	33	30.0%
	Two pregnancies	42	38.2%
	Three pregnancies	27	24.5%
	Four pregnancies	7	6.4%
	Five times or more	1	0.9%
	Total	110	100.0%
Number of children	None	2	1.82%
	One child	39	36.1%
	Two children	47	43.5%
	Three or more children	22	20.4%
	Total	110	100.0%
Number of abortions	None	72	65.5%
	One time	32	29.1%
	Two times	6	5.5%
	Total	110	100.0%
Mode of delivery	Normal vaginal delivery only	57	51.8%
	Caesarean section only	39	35.5%
	Both vaginal delivery and C-section	14	12.7%
	Total	110	100.0%

Participants demonstrated variable awareness of warning symptoms during pregnancy. Clinically significant but less commonly recognized symptoms, including convulsions (43.9%) and visual abnormalities (50.5%), were less often identified. A small proportion of respondents (4.5%) incorrectly identified food cravings as a warning symptom. Thirty-eight participants (8.9%) were unaware of any alarming symptoms during pregnancy.

Knowledge regarding harmful exposures during pregnancy was generally high. Smoking (94.4%) and alcohol (96.0%) were widely recognized as detrimental. However, fewer participants were aware of risks associated with consuming unpasteurized milk (47.4%) or soft cheeses (69.0%).

**Figure 1:**
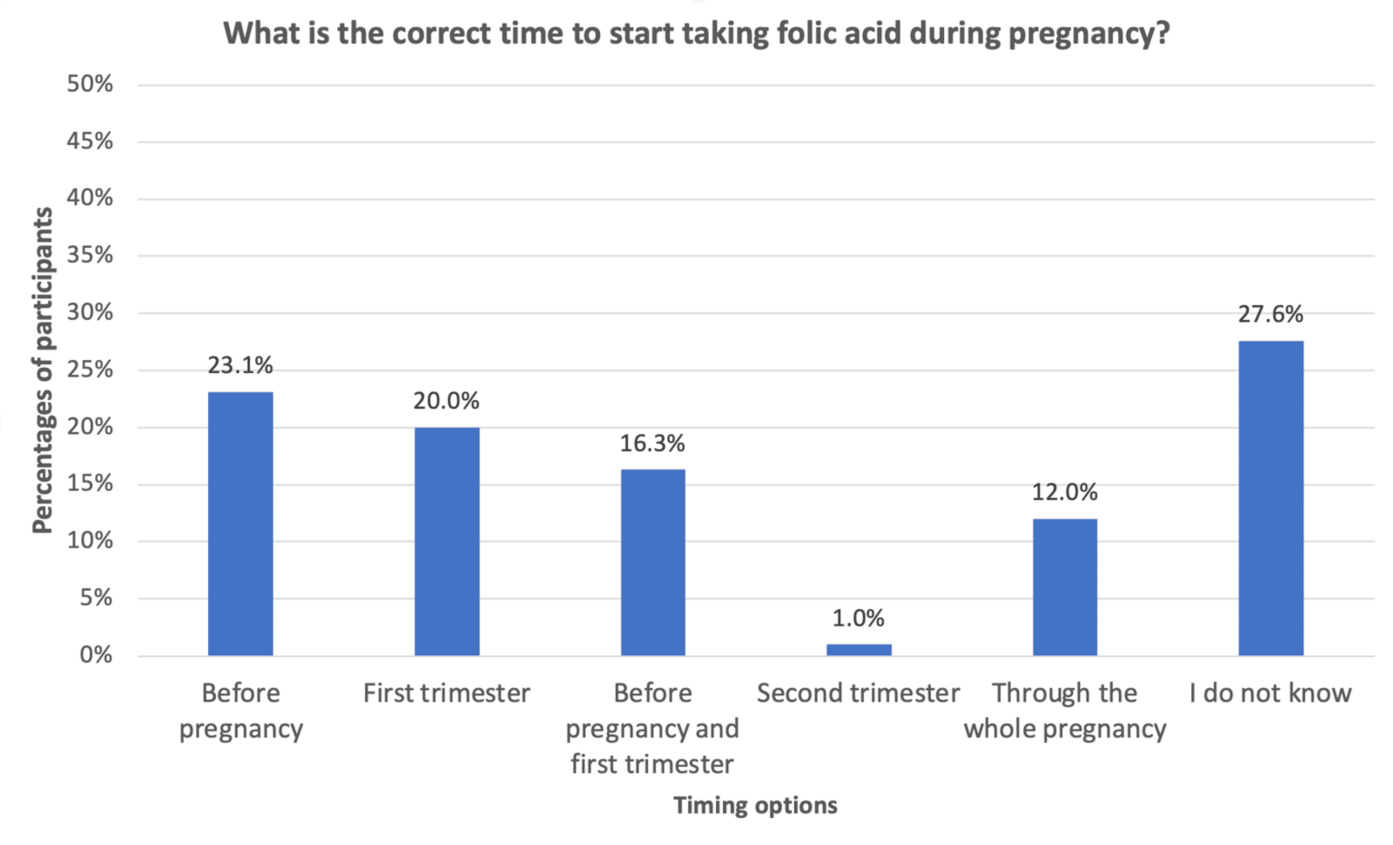
Knowledge of the appropriate time to start folic acid during pregnancy (n = 426).

Knowledge about the appropriate time to start taking folic acid during pregnancy was limited. Only 16.3% of participants correctly identified that folic acid should be taken before pregnancy and during the first trimester (Figure 1).

Knowledge and practices regarding antenatal care (ANC) visits

**Figure 2:**
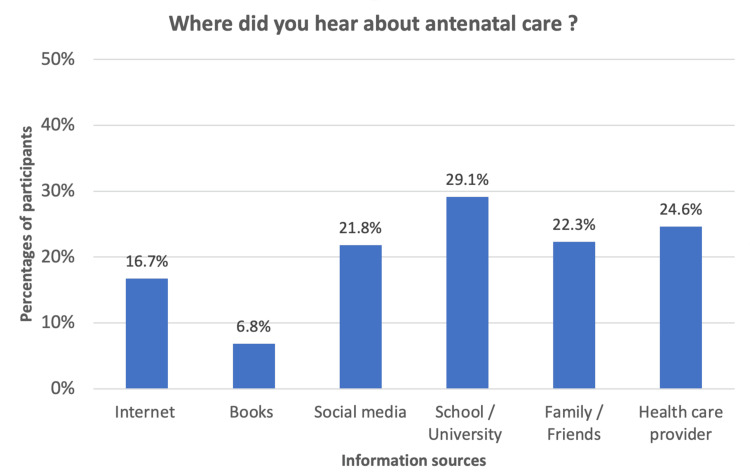
Sources of ANC information among participants (n = 426). Participants could select multiple sources. ANC: antenatal care.

Among all participants, 60.3% previously heard about ANC. Figure 2 illustrates the sources of their information.

The majority of participants demonstrated a good knowledge of ANC objectives. 94.1% recognize its importance for both maternal and fetal health. However, specific knowledge gaps were evident. High percentages correctly identified routine tests such as blood pressure monitoring (93.2%), blood glucose screening (91.5%), and urine testing (84.0%) as essential; a notable number incorrectly identified mammograms (25.8%), Pap smears (38.0%), and x-rays (19.0%) as needed during pregnancy.

Of the 110 participants who had been pregnant, 52 (47.3%) attended antenatal care visits, and among them, only 10.8% reported attending regularly. Half the participants knew that ANC visits should start in the first trimester. Additionally, only about one-third of the participants accurately reported the frequency of ANC visits recommended during each trimester.

**Figure 3:**
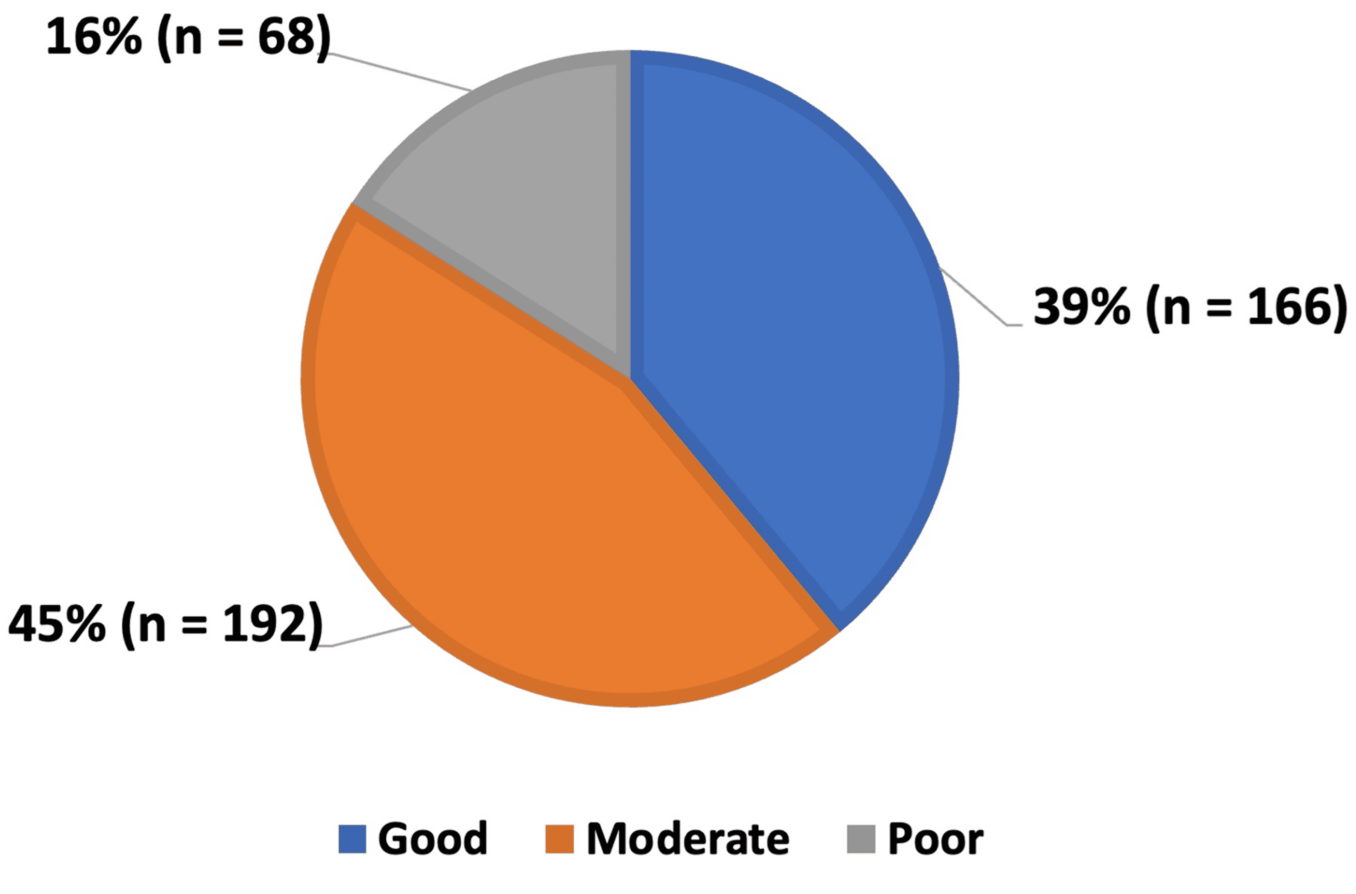
Level of knowledge about ANC among participants (n = 426). ANC: antenatal care.

After calculating each participant's total score, the knowledge level regarding ANC among women of childbearing age in the UAE was distributed as shown in Figure 3, with the majority of participants demonstrating good and moderate levels of knowledge. 

Correlation analysis revealed several significant associations between participants’ level of knowledge and key demographic variables. Education level demonstrated a strong association with knowledge (p = 0.015). Among participants with a college degree, 40.9% had good knowledge, compared with 35.2% among those with only a high school education, indicating that higher educational attainment is linked to better ANC awareness. Occupation was also significantly associated with knowledge (p = 0.001), with healthcare workers showing the highest proportion of good knowledge (82.9%), followed by women in non-healthcare occupations (42.0%), students (33.9%), and unemployed participants or housewives (29.3%). Gravidity showed a statistically significant association as well (p = 0.04), with multigravida women demonstrating higher levels of good knowledge (45.5%) than nulligravida (38.6%) and primigravida women (24.2%). Household income also correlated significantly with knowledge level (p = 0.007), as women from families earning above 25,000 AED reported the highest proportion of good knowledge (54.4%). Nationality was another significant factor (p = 0.035), though interpretation should be cautious due to distribution differences across groups.

In contrast, no significant associations were found between knowledge level and previous attendance at ANC (p = 0.586) or marital status (p = 0.462). However, married women demonstrated slightly higher rates of good knowledge (40.3%) than unmarried participants (38.0%). No statistically significant correlation was observed between age and knowledge (p = 0.07), although the 24-26 age group reported the highest proportion of good knowledge (58.2%).

## Discussion

The current study demonstrated that most women of childbearing age possessed either good or moderate levels of knowledge regarding ANC. Higher levels of knowledge were observed among participants with advanced education and multiple pregnancy experiences, compared to those pregnant for the first time. In contrast, age and marital status did not show a significant correlation with knowledge level. Overall, participants were aware of the purpose and services provided during ANC visits. However, notable knowledge gaps were identified regarding the appropriate timing of the first ANC visit and the recommended frequency of subsequent visits. Additionally, a few participants correctly recognized that folic acid should be taken both before conception and during the first trimester.

Strong evidence shows that adequate ANC attendance reduces complications during pregnancy and delivery [10-12]. Women who fail to receive adequate ANC have higher risks of perinatal complications, including preterm birth, reduced fetal movement, low birth weight, perinatal death, fetal distress, and increased neonatal intensive care unit (NICU) admissions [13]. This underscores the importance of ensuring that women of reproductive age possess sufficient knowledge about ANC and initiate care at the appropriate time.

A cross-sectional study conducted in the UAE in 2015 reported that Emirati women had relatively low levels of knowledge regarding pregnancy and ANC, highlighting the need for further education and awareness [14]. In contrast, the present study revealed that most women of childbearing age in the UAE demonstrated moderate and good knowledge of ANC. Notably, our sample included both Emirati participants (21.4%) and non-Emirati residents (78.6%), which may partly contribute to the difference between the studies.

Education level emerged as a significant predictor of ANC knowledge in our study, consistent with the findings of a research done in 2023 who reported that women with higher educational levels had better awareness regarding investigations conducted during pregnancy, dietary modifications, and folic acid intake. Moreover, a strong correlation was also demonstrated between ANC knowledge and socioeconomic status, with women from higher socioeconomic groups displaying greater awareness and healthier practices regarding diet and ANC visits [5]. Our findings align with this observation as participants from households with monthly incomes exceeding 25,000 AED had the highest proportion of good knowledge (54.4%). This reflects the fact that a higher socioeconomic status enhances access to healthcare services and facilitates higher education, positively influencing ANC knowledge. These patterns suggest that future public health efforts should prioritize women with lower educational attainment and lower socioeconomic status, as they consistently demonstrated lower levels of ANC knowledge in our sample. This association also identifies women with lower educational levels and lower socioeconomic status as a subgroup in whom knowledge gaps are more evident, which can help inform the design and direction of future educational and public health efforts.

Folic acid supplementation is essential during pregnancy, as it reduces the risk of abortion, supports fetal growth and development, and prevents congenital anomalies [15-17]. A study in Abu Dhabi found that while 79.1% of pregnant Emirati women had heard of folic acid and 66.7% were aware of its importance in preventing neural tube defects, only 7.8% reported using it before pregnancy. Approximately 10% were unaware of the correct timing for supplementation [18]. Our findings are consistent with this knowledge gap, as only 16.3% of participants correctly identified that folic acid should be taken before pregnancy and throughout the first trimester of pregnancy. This highlights the need for physicians and other healthcare providers to emphasize more strongly on the importance of folic acid intake.

In our study, a greater proportion of participants with good knowledge were UAE residents (84.8%) compared to UAE nationals. However, this disparity may be attributable to the uneven distribution of respondents, as UAE residents constituted 78.6% of the sample while UAE nationals accounted for only 21.4%. Therefore, this association should be interpreted with caution.

Limitations, strengths, and recommendations

This study used a non-probability convenience sampling method, which limits the ability to generalize the findings to the entire UAE population. In addition, data were collected only from public locations in Dubai, Sharjah, and Ajman, although these emirates host diverse communities, the sample may not fully represent women from regions not included in the data collection or those who do not frequently visit public venues. Therefore, the results should be interpreted with caution when considering nationwide applicability. The study also relied on self-reported information, which carries the possibility of recall or reporting bias.

Nevertheless, to our knowledge, no recent studies have evaluated baseline ANC knowledge among women of childbearing age in the UAE. This makes the present research a valuable contribution, providing insight into strengths and gaps in community awareness and serving as a foundation for future interventions. The relatively large sample size and analysis across multiple demographic and experiential variables further strengthen the study’s ability to identify patterns in ANC knowledge within the study population. Additionally, the inclusion of both Emirati and non-Emirati participants offers broader contextual insight into ANC awareness in the UAE’s diverse population.

Future research should consider probability-based sampling across all seven emirates and expanding geographic coverage and sampling strategies would help capture broader demographic variations and provide a more complete national perspective. Interventions such as awareness campaigns focusing on diet, physical activity, and folic acid supplementation during pregnancy, as well as improved accessibility of ANC services, particularly for women from lower-income backgrounds, are to address areas where knowledge remains insufficient. Moreover, clinical practice should incorporate stratified educational protocols addressing identified knowledge deficits, particularly regarding supplement timing and warning signs of pregnancy. Such efforts could enhance ANC awareness and improve maternal and neonatal health outcomes across the UAE community. 

## Conclusions

This study aimed to assess the level of knowledge about ANC among women of childbearing age in the United Arab Emirates and found that participants generally demonstrated a moderate and good level of knowledge. Higher education levels, healthcare-related occupations, higher family income, and multiple previous pregnancies were associated with higher knowledge. At the same time, notable gaps remained in areas such as diet, exercise, and the timely initiation of folic acid supplementation. These findings highlight that although overall understanding of ANC is present, there is a need for greater emphasis on the practical aspects of ANC to ensure this knowledge translates into consistent and effective practice.

The study was limited by its non-probability sampling method and the underrepresentation of UAE nationals, which may affect generalizability. Nonetheless, the insights gained are relevant for guiding maternal health initiatives in the region. Future research should build on this work by using larger and more representative samples and consider longitudinal or qualitative designs to explore the reasons behind persistent misconceptions more effectively. Overall, this study provides valuable local data that can inform future efforts to enhance maternal and neonatal health outcomes in the UAE.

## Appendices

Appendix A 

**Table 3:** English version of the questionnaire. This questionnaire was developed by the authors of this study after reviewing the literature about the topic. The Arabic version of the questionnaire is available upon reasonable request from the corresponding author.

Section name	Item number	Questions/instructions	Choices
Demographics	A1	Are you a female above the age of 18 years and living in the United Arab Emirates?	Yes
			No
	A2	Please write the last three digits of your phone number:	Open-ended
	A3	Please write the last three letters of your name:	Open-ended
	A4	What is your age in years?	18 to 20
			21 to 23
			24 to 26
			27 to 29
			30 to 32
			33 to 35
			Above 35
	A5	What is your nationality?	UAE national
			Arab resident
			Non-Arab resident
	A6	Which of the following best describes your occupation?	Student
			Housewife
			Currently employed in a healthcare sector
			Currently employed in a non-healthcare sector
			Previously employed in a healthcare sector
			Previously employed in a non-healthcare sector
			Unemployed
	A7	What is the highest educational level you have completed?	No schooling
			Primary school
			Middle school
			High school
			Technical/Trading school
			Diploma
			Bachelor’s
			Post-graduate
	A8	What is your marital status?	Single
			Married
			Engaged
			Divorced
			Widowed
	A9	Which of these describes your average family monthly income?	Less than 4,000 AED
			4,000-9,999 AED
			10,000-14,999 AED
			15,000-19,999 AED
			20,000-24,999 AED
			More than 25,000 AED
			Prefer not to answer
Obstetric history	B1	How many times have you been pregnant?	Never
			One time
			Two times
			Three times
			Four times
			Five or more
	B2	How many children do you have?	None
			One
			Two
			Three or more
	B3	How many times you had abortion/s?	None
			One
			Two
			Three or more
	B4	What was the mode of your delivery/s?	Normal vaginal
			C-section
			Both
	B5	What was the place of delivery/s? (Select all that apply)	Governmental hospital
			Private hospital
			Home
Antenatal care practices	C1	Have you ever heard about antenatal care?	Yes
			No
	C2	Where did you hear about antenatal care? (Select all that apply)	Doctor/Healthcare provide
			Internet
			Family/Friends
			School/University
			Social media
			Books
	C3	Did you go to antenatal care visits before?	Yes
			No
	C4	Were you regular in your antenatal care visits?	Yes
			No
			I do not remember
Knowledge about antenatal care and pregnancy	D1	To whom do you think the purpose of antenatal care visits are?	For mother
			For baby
			Both (mother and baby)
			I do not know
	D2	Which of the following services are provided during antenatal care?.	Each service had the following response options: Yes, No, I do not know
		Education about nutritional needs of pregnant women.	
		Counseling and education about pregnancy complications.	
		Education about delivery methods.	
		Education about breastfeeding.	
		Providing the necessary vaccinations to pregnant women.	
		Providing supplements for pregnant women.	
	D3	Rate your level of agreement to the following statements.	Likert scale (strongly agree, agree, neutral, disagree, strongly disagree)
		Pregnant women need to go for regular antenatal check-ups.	
		Antenatal check-ups detect complications for mother and fetus.	
		Antenatal care reduce maternal and neonatal death.	
		Pregnant women can do any exercises.	
		Pregnant women can gain as much weight as they want.	
		Pregnant women should eat portions of two people.	
		Home delivery is better than hospital delivery.	
		Pregnant women can take any medication.	
	D4	At what time pregnant women start going to antenatal care visits?	During the first three months
			During the first six months
			Only if there is an issue
			No need to visit
			I do not know
	D5	How often should a pregnant woman go for antenatal care visits between the first and third month?	Every week
			Every two weeks
			Every four weeks
			Twice a week
			No need to visit
			I do not know
	D6	How often should a pregnant woman go for antenatal care visits between the fourth and eighth month?	Every week
			Every 2 weeks
			Every 4 weeks
			Twice a week
			No need to visit
			I do not know
	D7	How often should a pregnant woman go for antenatal care visits from the ninth month until delivery?	Every week
			Every two weeks
			Every four weeks
			Twice a week
			No need to visit
			I do not know
	D8	Does a pregnant woman need to undergo the following tests during pregnancy?	Each service had the following response options: Yes; No; I do not know
		Weight and height measurement	
		Blood pressure monitoring	
		X-rays	
		Ultrasound	
		Blood sugar level	
		Mammogram	
		Urine test	
		Pap smear	
		Screening for Hepatitis-B virus	
		Screening for HIV virus	
	D9	What is considered a full term pregnancy?	Less than 37 weeks
			Between 37-42 weeks
			More than 42 weeks
			I do not know
	D10	In your opinion, where should a pregnant woman deliver her baby?	Health care facility
			Home
			I do not know
	D11	What are the alarming signs during pregnancy? (Select all that apply)	Excessive vomiting
			Persistence swelling of hands and feet
			Vaginal bleeding
			Decrease/no movement of the baby
			Food cravings
			High blood pressure
			High blood glucose level
			Convulsions
			Abdominal pain
			Visual disturbance
			I do not know
	D12	Which of the following is harmful to consume during pregnancy? (Select all that apply)	Smoking
			Alcohol drinking
			Unpasteurized milk
			Raw fish
			Dairy products
			I do not know
	D13	Do you think pregnant women need to take folic acid supplements?	Yes
			No
			I do not know
	D14	What is the timing to start taking folic acid	Before pregnancy
			First trimester
			Before pregnancy and first trimester
			Second trimester
			Through the whole pregnancy
			I do not know
